# Cardioprotective and Antihyperlipidemic Effects of *Arthrospira maxima* Phycobiliproteins in a Prediabetic Rat Model of Acute Myocardial Infarction

**DOI:** 10.1021/acsomega.5c05120

**Published:** 2025-10-07

**Authors:** Van Dan Castro-Gerónimo, Alberto Sánchez-Medina, Germán Alberto Chamorro-Cevallos, Candelaria Galván-Colorado, Israel Ramírez-Sánchez, Enrique Méndez-Bolaina, Rosa Virginia García-Rodríguez

**Affiliations:** † Centro de Investigaciones Biomédicas, Doctorado en Ciencias Biomédicas, 27870 Universidad Veracruzana, Av. Dr. Luis Castelazo Ayala S/N, Col. Industrial Ánimas, CP., Xalapa, Veracruz 91190, Mexico; ‡ Instituto de Química Aplicada, Laboratorio de Farmacología y Quimiometría, Universidad Veracruzana, Av. Dr. Luis Castelazo Ayala S/N, Col. Industrial Ánimas, CP., Xalapa, Veracruz 91190, Mexico; § Escuela Nacional de Ciencias Biológicas, Laboratorio de Toxicología Preclínica, Departamento de Farmacia, 27740 Instituto Politécnico Nacional, Av. Wilfrido Massieu s/n, Ciudad de México, CDMX 07738, Mexico; ∥ Escuela Superior de Medicina, Sección de Estudios de Posgrado e Investigación, Instituto Politécnico Nacional, Salvador Díaz Mirón esq. Plan de San Luis S/N, Miguel Hidalgo, Casco de Santo Tomás, Ciudad de México, CDMX 11340, Mexico; ⊥ Maestría en Ciencias en Procesos Biológicos, Universidad Veracruzana, Prolongación de Oriente 6 #1009, Col. Rafael Alvarado, CP., Orizaba, Veracruz 94340, Mexico

## Abstract

*Arthrospira maxima* (Am) is a filamentous cyanobacterium with multiple nutraceutical components, such as essential amino acids, proteins, secondary metabolites, and pigments, such as β-carotene and phycobiliproteins. Am phycobiliproteins are water-soluble proteins and accessory pigments involved in the photosynthetic process with multiple beneficial health effects, such as antiviral, anticancer, and antioxidant. The aim of this work was to analyze the cardioprotective and antihyperlipidemic effects of Am and Am phycobiliproteins ((ExPhy) containing 76% C-phycocyanin (C-PC), 18.4% allophycocyanin (APC), and 3.8% phycoerythrin (PE)) in an acute myocardial infarction model. Methods: Prediabetic Wistar rats were administered with ExPhy (6.75 mg/kg, 12.50 mg/kg, 75 mg/kg) and Am (500 mg/kg) for 21 days, then the ischemia/reperfusion model was performed (1/4) measuring infarct area vs healthy area, in addition to biochemical lipid tests. Results: A reduction in cardiac necrotic damage produced by ischemia was found, as well as a reduction in total cholesterol and LDL cholesterol without significant differences in triglycerides and HDL; however, a reduction in the atherogenic index was observed in the doses of ExPhy 12.50 mg/kg and Am 500 mg/kg administered subchronically. Conclusion: Am and ExPhy showed cardioprotective and antihyperlipidemic action in the prediabetic rat model, because of its ability to modulate key signaling pathways involved in cell survival, inflammation, and lipid metabolism, hence acting as potential adjuvants against damage caused by cardiac ischemia and reperfusion.

## Graphical abstract

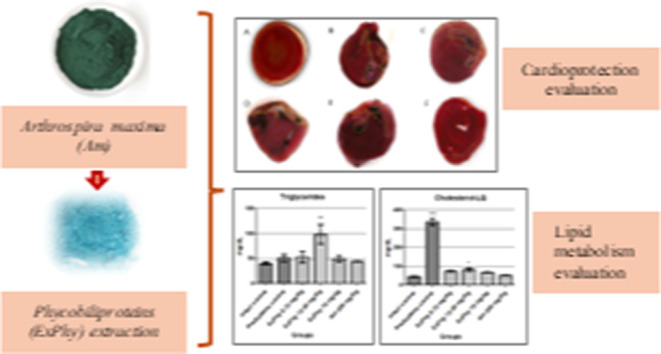

## 1 Introduction

*Arthrospira maxima* (Am) is a cyanobacterium used as a dietary supplement because of its high nutrient content such as proteins (phycobiliproteins), minerals, vitamins, fatty acids, and β-carotene.^1^ It has been proven at an experimental level in vivo and in vitro, to possess antiviral, antiulcerogenic, anticarcinogenic, anti-inflammatory, and cardioprotective properties, as well as hypocholesterolemic, hypoglycemic, and antihypertensive effects.^2^

Prediabetes is a metabolic state prior to diabetes *mellitus* (DM) and is defined as a disorder of carbohydrate metabolism in which blood glycemic levels do not reach the values of established diabetes but are above normal values.^1,3^ Its occurrence predisposes to a 1.5 times higher risk of cardiovascular disease compared to a person with normal glycemic indices, indicating that hyperglycemia is directly implicated with cardiovascular risk. This metabolic condition is accompanied by numerous vascular complications such as diabetic cardiomyopathy, resulting in pathophysiological processes in the coronary microcirculation due to changes in glycemic and lipid metabolism.^4,5^

First-line treatments to treat cardiometabolic risk focus on thrombolytic mechanisms such as acetylsalicylic acid and hypoglycemic agents such as glucagon-like peptide-agonists and metformin; however, being a chronic condition, therapeutic adherence to drug treatment coupled with adverse drug reactions and side effects make its management a challenge in the biomedical field.^6^ Hence, it is important to develop and evaluate complementary treatments that are easily accessible and cost-effective such as rich phycobiliprotein extracts of *Arthrospira maxima*. Those can attenuate the clinical signs and symptoms of prediabetes and combat complications such as the risk of atherosclerotic disease and acute myocardial infarction.

It is known that Am phycobiliproteins act by modulating antioxidant enzymes in cells, which inactivate chemical species such as ROS and NOS, increasing the activity of SOD and CAT; likewise, this microalga shows enhanced activity when subjected to additional factors that induce oxidative stress by inhibiting lipid peroxidation and preventing DNA damage, resulting in an increase in cell viability in tissues subjected to oxidative cellular stress. These properties have been attributed to phycobiliproteins, which are divided into three groups: phycoerythrin, C-phycocyanin, and allophycocyanin. The pharmacological activity of C-phycocyanin (C-PC), the main phycobiliprotein of blue-green algae, is due to the antioxidant activity of phycocyanobilin, which is the apoprotein responsible for interacting with unstable species, as well as for the inhibition of microsomal lipoperoxidation because of its interaction with peroxyl radicals.^7,8^ The aim of the present study is to evaluate the cardioprotective and antihyperlipemic effect of *Arthrospira maxima* phycobiliproteins (ExPhy), administered for 21 days in prediabetic male Wistar rats in the model of acute myocardial infarction (ischemia/reperfusion 1/4). The ExPhy used in this study was determined to contain 76% C-phycocyanin (C-PC), 18.4% allophycocyanin (APC), and 3.8% phycoerythrin (PE).^9^ After treatment, biochemical markers, including total cholesterol, triglycerides, HDL, LDL, atherogenic index and cardioprotective effect (percentage of infarcted area/healthy area) were assessed.

## 2 Results

### 2.1 Ischemia/Reperfusion Model in Prediabetic Rats

The 1/4 ischemia/reperfusion model was performed in animals with prediabetes from the following groups (Figure 1): intact control (1A), prediabetes control (control +) (1B), ExPhy 6.75 mg/kg (1C), ExPhy 12.50 mg/kg (1D), ExPhy 75 mg/kg (1E), and Am 500 mg/kg (1F), observing different degrees of necrotic injury, delimited by yellow lines.

**Figure 1:**
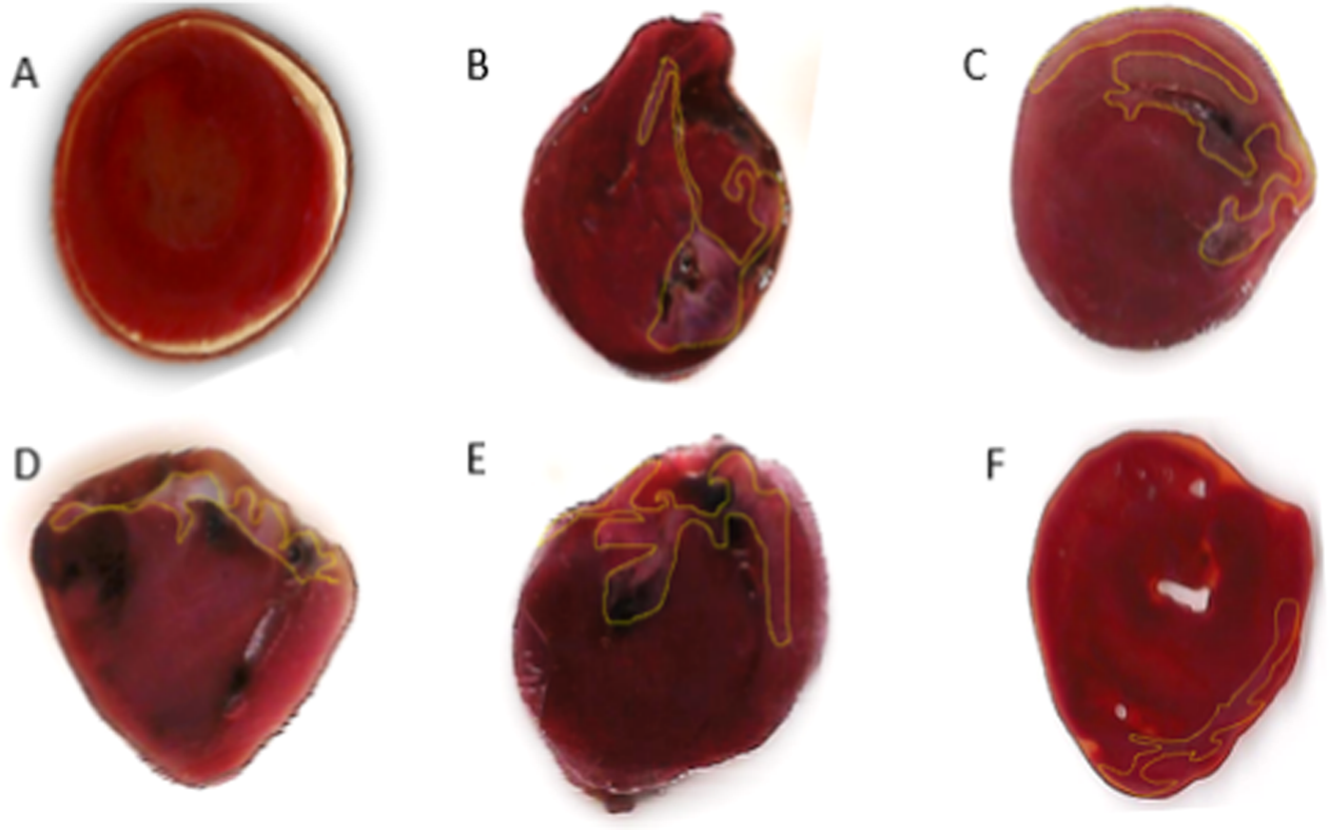
Effects of ExPhy on total infarct area. (A) Intact control. A uniform area without morphological changes is seen. (B) Prediabetes control. An extensive necrotic area is seen. (C) ExPhy 6.75 mg/kg. (D) ExPhy 12.50 mg/kg. (E) ExPhy 75 mg/kg. (F) Am 500 mg/kg. A reduction in the cardiac area damaged by ischemia is observed. Photos were taken by the authors.

The cardioprotective effect of ExPhy and Am treatment is shown in Figure 2. The intact control did not show necrotic lesions. In the control group, (+) ischemic damage produced by acute infarction is observed (Figure 1B). Treatment with ExPhy (6.75 mg/kg, 12.50 mg/kg, and DE_50_) as well as Am 500 mg/kg (Figure 1C–F, respectively) showed a reduction in the damage produced by the ischemia/reperfusion process.

**Figure 2:**
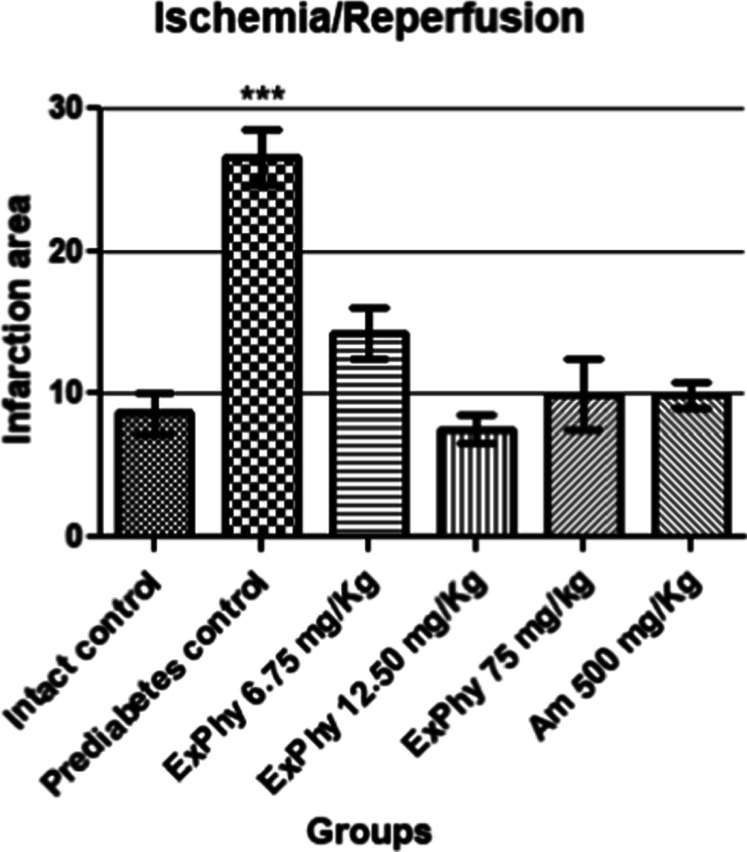
ExPhy and Am pretreatments followed by ischemia/reperfusion injury, exerts cardio-protection on myocardial tissue, lowering total infarction area, compared to the prediabetes control. *** indicates *p* ≤ 0.001. Data are expressed as the mean ± SEM (*n* = 5).

Figure 2 shows the 1-way ANOVA analysis of the ischemia/reperfusion data obtained. The percentage of infarct area of the different treatments is observed, with clear differences between prediabetes control and all experimental groups (*p* < 0.001).

### 2.2 Lipids Determination

Figure 3 shows lipid biochemical parameters such as total cholesterol (Cholesterol-LQ) in [Fig fig3]a, high density cholesterol (HDLc-P) in [Fig fig3]b, low density cholesterol (LDL-c) in [Fig fig3]c, and triglycerides (GPO-POD) in [Fig fig3]d after 21 days of treatment.

**Figure 3:**
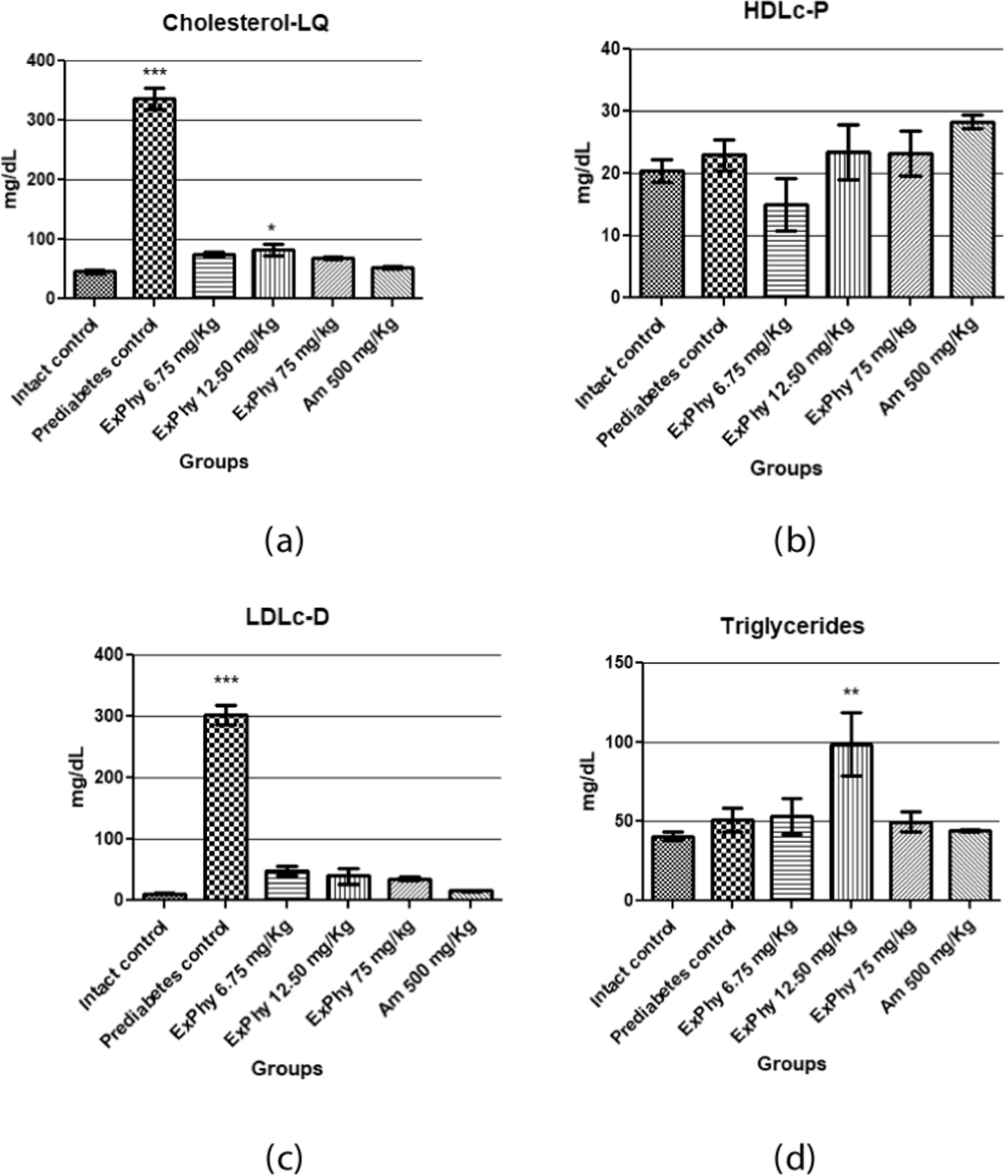
Determination of serum lipids. (a) Determination of total cholesterol. A significant reduction is observed in all doses of ExPhy and Am vs prediabetes control (*p* ≤ 0.05). (b) Determination of HDL cholesterol. No significant differences were seen between the treatment and prediabetes control groups. (c) Determination of LDL cholesterol. A significant decrease is observed for all ExPhy and Am treatments vs prediabetes control (*p* ≤ 0.001). (d) Determination of triglycerides. An increase is seen in the ExPhy 12.50 mg/kg dose vs the other doses administered and the intact and prediabetic controls (*p* ≤ 0.01) (*n* = 5 for all groups).

The atherogenic index was calculated using Castelli’s formula (total cholesterol/HDL cholesterol) as a predictor of cardiovascular risk (Figure 4).

**Figure 4:**
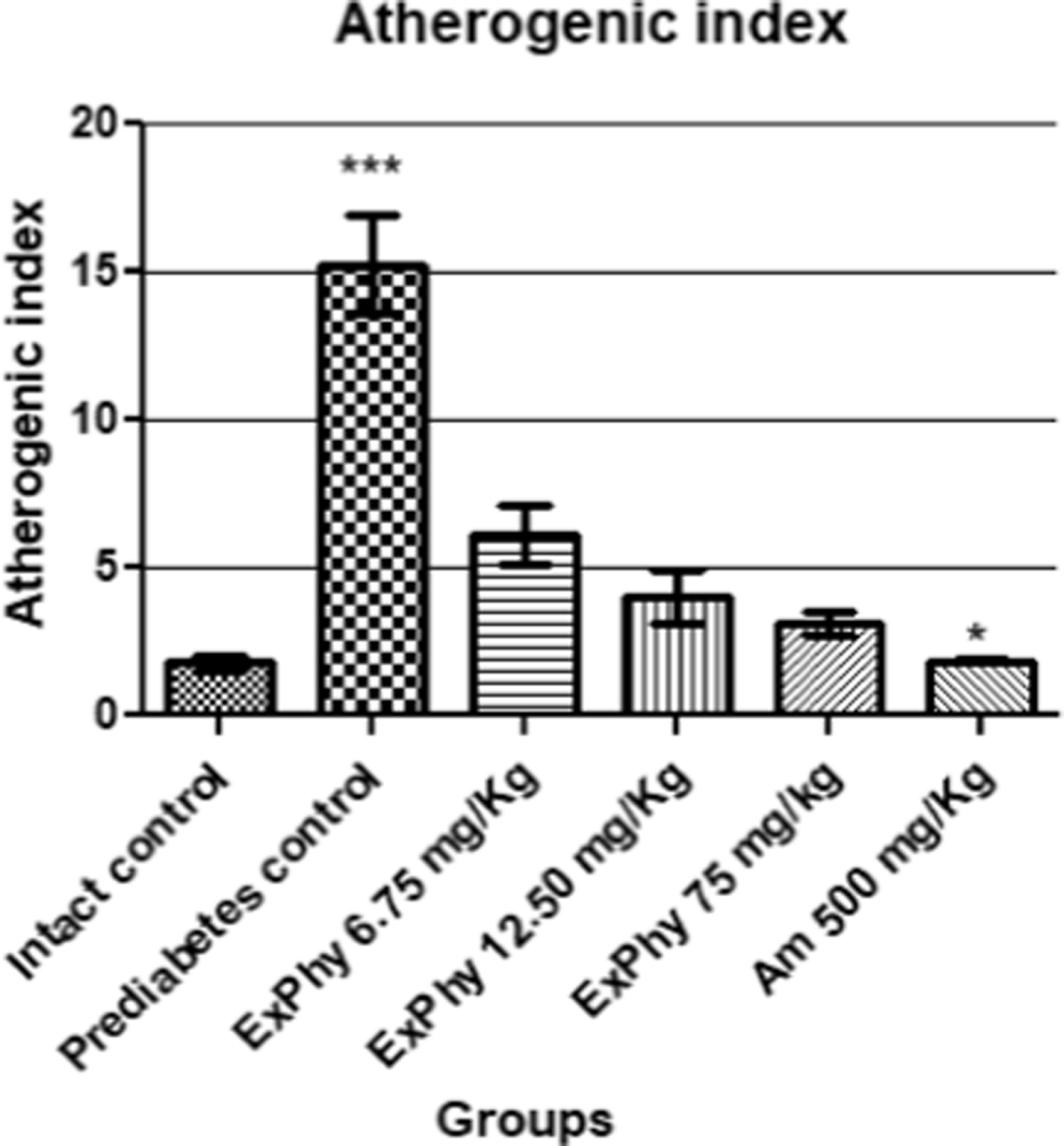
Calculation of the atherogenic index. A significant reduction in atherogenic risk is seen for all ExPhy and Am treatment schemes vs prediabetes control (*p* ≤ 0.001).

## 3 Discussion

### 3.1 Ischemia/Reperfusion Model in Prediabetic Rats

The acute infarction model was tested in prediabetic conditions. There was a significant reduction in the percentage of infarcted areas in the groups administered with ExPhy and Am. When the histology was observed using 1% triphenyltetrazolium chloride, the reduction of the myocardial tissue necrotic area can be seen when the different treatment schemes were applied, especially in the ExPhy 12.50 mg/kg group, which reversed the necrosis produced by the ischemia process.

The establishment of DM generates various complications such as coronary heart disease, acute myocardial infarction, peripheral arterial disease, diabetic retinopathy, and kidney disease, among others.^8,10^ The most serious vascular complications of DM and prediabetes are diabetic macro- and microangiopathy, which increase the risk of coronary artery disease, peripheral arterial disease, and cerebrovascular disease, all associated with the high atherogenic risk in this pathological condition.^11^ Arterial walls are vulnerable in DM, due to the presence and accumulation of fibrous layers, lipid deposition and calcification in the intima, neovascularization of plaque in the intima, hypercoagulability, atheromatous intraplaque hemorrhage, and dysfunctional endothelial repair. These complications lead to the maintenance of vascular damage by neovascularization called *vasa vasorum,* which is a response to hypoxia and localized inflammation. The new blood vessels, being immature and weak, are prone to rupture, generating new inflammatory and ischemic events and leading to diabetic cardiomyopathy. This pathological spectrum includes multiple mechanisms, including glucotoxicity and lipotoxicity, chronic inflammation, structural myocardial changes (fibrosis), and coronary microvascular dysfunction, which leads to periarterial fibrosis, arteriolar thickening, focal constrictions, microvascular tortuosity, capillary membrane thickening, and reduced capillary density, as well as deficit of endothelium-dependent coronary vasodilation.^12,13^ The preservation of endothelial function and the correct balance of oxidative stress through the consumption of exogenous antioxidants have been suggested as an optimal strategy for the prevention of vascular complications in DM.^14^

It has been reported that the administration of pigments such as bilirubin, biliverdin, and phycobilins such as C-PC (300 mg/kg for 10 weeks) protects against diabetic complications such as albuminuria and renal damage, in addition to normalizing circulating values of TGB-β and fibronectin, decreasing vascular inflammation. The results obtained with respect to Am administration are based on the evidence that Am supplementation at a dose of 250 mg/kg reduces the global longitudinal tension of the left ventricle in ischemic events, having cardioprotective effects against cardiac damage produced in acute myocardial infarction.^15^ The cardioprotective effects of Am observed in this model are consistent with those reported in the literature, by decreasing systolic pressure, vascular inflammation markers such as sVCAM-1, sE-selectin, and endothelin-1 levels, resulting in a decrease in endothelial damage.^16^ Additionally, the administration of phycobiliproteins significantly reduces oxidative stress values by mediating NADPH oxidase activity, an endogenous source of ROS such as superoxide.^17,18^ This antioxidant response is partly regulated via modulation of the Nrf2/ARE pathway, which leads to upregulation of cytoprotective enzymes such as heme oxygenase-1 (HO-1), glutathione peroxidase, and superoxide dismutase, enhancing cellular resilience against oxidative injury during ischemia/reperfusion.^19^ It also modulates the activity of enzymes such as lactate dehydrogenase and creatinine kinase in the coronary effluent, decreasing infarct size and improving the recovery of cardiac function. In addition, it suppresses the generation of free radicals by ischemia/reperfusion by reversing the expression of p38, Bax, Caspase-3, and Bcl-2, which are indicators of apoptotic activity.^20^ These changes are closely related to the inhibition of the mitochondrial apoptosis pathway and stabilization of mitochondrial membrane potential, preserving cardiomyocyte integrity during reperfusion injury.^21^ These are the most accepted mechanisms of action for the cardioprotective action observed in preclinical models, reducing macroangiopathic changes and diabetic atherosclerosis; highlighting the results obtained, the treatment of phycobiliprotein extract, as well as *Arthrospira*, effectively reverse the damage produced by surgical ischemia in the model of acute myocardial infarction due to its antioxidant activity, promoting cell viability in an ischemia/reperfusion event. Moreover, the improvement in endothelial function may be explained by enhanced nitric oxide (NO) bioavailability, through inhibition of eNOS uncoupling and prevention of peroxynitrite formation, mechanisms essential to preserving coronary microvascular perfusion.^22^ This positions the treatment as a novel therapy against complications occurring after an atherothrombotic event, which will prevent future complications, such as adverse cardiac remodeling. The reduction of total heart damage results in increased cell viability after a myocardial ischemic event, which in the clinic means a benefit in the quality of life of patients who suffer an infarction by reducing clinical signs and symptoms when developing new therapies to preserve cardiac structure and function after an infarction.^23^

### 3.2 Biochemical Analysis in the Prediabetes Model

Dysregulation of total cholesterol, triglycerides, and LDL-c and HDL-c levels in DM facilitates the atherosclerotic process, which in combination with the cellular oxidative stress caused by the hyperglycemic state generates a spectrum of conditions that increase cardiometabolic risk, due to a mobilization of free fatty acids from peripheral deposits, released by insulin-sensitive lipases, which end up being interconverted to phospholipids and cholesterol.^24^ The data obtained with respect to blood glucose show a significant decrease in the treatment with Am (500 mg/kg) after 21 days of administration. These data agree with the antihyperlipemic activity previously reported by several authors analyzed by meta-analysis, in which a reduction in circulating lipids such as triglycerides, total cholesterol, LDL cholesterol, and VLDL and a significant increase in HDL after the administration of cyanobacteria, effect attributed to its antioxidant capacity, which can reduce oxidative damage to pancreatic β-cells in prediabetic and diabetic state.^25–27^ At the molecular level, this is associated with decreased expression of proinflammatory cytokines such as TNF-α and IL-6, reduction in NF-κB signaling, and downregulation of genes involved in hepatic lipogenesis, such as SREBP-1c and ACC1, contributing to improved lipid handling and insulin sensitivity.^28^

On the other hand, it has been reported that the administration of a treatment scheme like the one proposed in this work reduces the impacts of glucotoxicity by the presence of DM, reduces serum glucose, glycosylated hemoglobin, and MDA levels, and increases serum insulin. Likewise, it induces the antioxidant activity of endogenous enzymes and normalizes their expression by mRNA, inducing the expression of the pyruvate carboxylase enzyme, CASP-3 by activating the MAPK pathway.^29^ This pathway is critical for regulating cell survival, stress responses, and insulin signaling in target tissues, contributing to improved glucose uptake and metabolic control.^30^ Because of this, Am administration protects from the deleterious effects of dysglycemia and dyslipidemia through its antioxidant, anti-inflammatory, and antiapoptotic activity. This agrees with the data obtained in the glycemic and lipid profile performed, in which the *Arthrospira* scheme clearly reduced total cholesterol, triglycerides, and LDL cholesterol and increased HDL cholesterol, although without reaching statistical significance. As a result, the atherogenic risk was the lowest with respect to all of the phycobiliprotein extract treatments.

On the other hand, it has been reported that the administration of C-PC isolated from *Arthrospira platensis* modulates the hepatocyte gluconeogenic response through the enzyme phosphoenolpyruvate carboxykinase and glucose-6-phosphatase. In addition, it improves the cellular sensitivity to glucose. These effects are mediated by the activation of different intracellular pathways such as IRS/PI3K/Akt and SIRT1/LKB1/AMPK, which are involved in gluconeogenesis, glycolysis, and lipolysis. The activation of the AMPK pathway promotes fatty acid oxidation, inhibits hepatic gluconeogenesis, and enhances glucose transporter (GLUT4) translocation in muscle tissue, resulting in improved glycemic control. Meanwhile, the SIRT1 axis modulates mitochondrial biogenesis and oxidative metabolism, which may explain the improvements in energy balance and metabolic homeostasis seen with treatment.^31^ Therefore, C-PC treatment is a promising treatment in combating cellular complications such as reticular stress, mitochondrial dysfunction, and lysosomal dysfunction.^32^ The above-reported observation partially agrees with the data obtained in the glycemic profile of prediabetic subjects because only the administration of phycobiliprotein extract at 6.75 mg/kg reduced the glucose (data not shown), although without reaching statistical significance. Regarding the lipid profile, all treatments clearly reduced the values of total cholesterol and triglycerides, except for the 12.50 mg/kg group, which increased this parameter probably due to the release of free fatty acids from the adipose tissue to the bloodstream. Regarding HDL cholesterol values, there was a nonsignificant reduction in the 6.75 mg/kg group with respect to the experimental groups, and for LDL cholesterol, there was again an extremely significant reduction in all the groups administered with ExPhy. With the lipid profile, the atherogenic risk index was obtained, which was drastically reduced in the DE_50_ group with respect to the prediabetic control, with values like those administered with Am 500 mg/kg, offering effective cardioprotection in acute myocardial infarction in comorbidity with the presence of prediabetes.

It has been reported that *Arthrospira* supplementation is effective in reducing plasma lipids from doses of 100 mg/kg for 28 days with a subsequent decrease in the atherogenic index, improving the prognosis of cardiovascular disease. This is mainly accomplished by several mechanisms of action comprising fecal excretion of cholesterol and bile by reducing cholesterol solubilization and poor absorption in the intestine, weight loss by reducing macrophage infiltration to visceral fat, prevention of hepatic fat accumulation, improvement in insulin sensitivity, and improvement in satiety by increasing leptin resistance in the arcuate nucleus.^33,34^ On the other hand, *Arthrospira* is known to contain H-b2 glycolipid, which inhibits pancreatic lipase activity in a dose-dependent manner, reducing postprandial triglyceride levels, which has also been proven by C-PC administration. Finally, it has been reported that it helps in the prevention of cholesterol accumulation by gamma linolenic acid, together with the niacin content, which improves dyslipidemia. By all these mechanisms, the administration of Am and C-PC improves dyslipidemia, obesity, and weight loss, suggesting a protective role in the prevention of atheromatous plaque formation and the consequent reduction of cardiometabolic risk, improving endothelial dysfunction, hypertension, and atherosclerosis.^9,35,36^

Although the prediabetic rat model with acute myocardial infarction is widely used and clinically relevant, it does not fully reproduce the complexity of human pathophysiology. Furthermore, future studies involving transcriptomic analyses, proteomic analyses, or pharmacodynamic assays would contribute to understanding the molecular mechanisms involved in the cardioprotective and antihyperlipidemic effects.

## 4 Conclusions

This study aimed to evaluate the cardioprotective and hypolipidemic effects of *Arthrospira maxima* and its phycobiliproteins in an acute ischemia and reperfusion model using male Wistar rats. The administration of the *Arthrospira maxima* extract and its phycobiliproteins demonstrated significant cardioprotective effects in the acute ischemia and reperfusion model, which can be attributed to their ability to scavenge reactive oxygen species, inhibit lipid peroxidation, and mitigate oxidative stress-induced damage. Additionally, *Arthrospira maxima* supplementation showed a notable hypolipidemic effect in experimental animals, indicating its potential in reducing the risk of atherosclerosis and cardiovascular diseases associated with dyslipidemia.

Furthermore, the findings of this study provide insights into the underlying mechanisms responsible for the observed effects. *Arthrospira maxima* and its phycobiliproteins were found to modulate key signaling pathways involved in cell survival, inflammation, and lipid metabolism. These molecular pathways include the activation of antioxidant enzymes, inhibition of proinflammatory cytokines, and regulation of lipid metabolism-related genes.

Overall, these findings support the potential use of *Arthrospira maxima* as a natural therapeutic agent for the prevention and treatment of cardiovascular diseases. Further studies could contribute to exploring the translational potential of these findings in human clinical trials.

## 5 Materials and Methods

### 5.1 Extraction of Phycobiliproteins (ExPhy)

*Arthrospira maxima* (Am) was obtained from the commercial brand Spiral Springs in a 250 g fine powder presentation. For phycobiliprotein extraction (ExPhy), an aliquot of 5 g was weighed on an analytical balance (Ohaus Pioneer) and resuspended in phosphate buffered saline solution (PBS 25 mM, pH 7.4) with mechanical stirring.

Subsequently, it was frozen in liquid nitrogen (−196 °C) for 2 h, and after that time, it was thawed in a water bath (30 °C) for 30 min. It was ultracentrifuged at 10,000 rpm at 4 °C (Hermle Labortechnik) in 1.5 mL tubes (Eppendorf) for 30 min, the blue supernatant was recovered, and the collection was again subjected to the previous centrifugation cycle.

The collected supernatant was recovered in 15 mL tubes (Falcon) protected from light, and spectrophotometer readings were taken to calculate the separation factor, measured as the absorbance ratio *A*_620_/*A*_650_ with 480 μL of PBS and 20 μL of phycobiliprotein supernatant. The sample was stored in a horizontal freezer (Frigidaire) at −20 °C. Subsequently, the sample was freeze-dried (Labconco FreeZone) at −50 °C and 0.0133 mbar for 36 h until a fine light blue powder was obtained.^37,38^ In the present study, the extract used of Am (ExPhy) was previously characterized by Galvan-Colorado et al.,^9^ showing a 2.25% phycobilin C-PC content.

### 5.2 Animals

Male rats of the Wistar strain, with an initial weight of 250 g at the beginning of the treatments, were distributed in 5 groups (*n* = 5) as follows: Intact control and Prediabetes control: ExPhy 6.75 mg/kg, ExPhy 12.50 mg/kg, ExPhy 75 mg/kg, and Am 500 mg/kg p.o. Animals were feed with commercial diet (Nutricubos Purina, México). Subjects were maintained under controlled conditions of a 12/12 h light/dark cycle. The experimental subjects were obtained from the Biotherium of the Faculty of Chemical Sciences, Orizaba campus, and the maintenance and performance of experimental procedures were in accordance with the Manual of Recommended Procedures for Animal Research in the Cardiovascular Pharmacology Laboratory (USA, National Research Council) and under the technical specifications for the Mexican Official Standard’s production, care, and use of laboratory animals (NOM-062-ZOO-1999). The study protocol was reviewed and approved by the Research and Bioethics Committee of the Faculty of Chemical Sciences at Universidad Veracruzana under registration number B/059/2020.

### 5.3 Prediabetes Model

Prediabetes in the experimental subjects was induced with streptozotocin (STZ) in a single dose. STZ was prepared in sodium citrate solution, 10 mM, pH 4.5. Individuals weighing approximately 100 g were given a dose of 33 mg/kg i.p. After administration, glucose values were measured on 3 occasions after reaching 125, 150, and 200 g of weight. Animals who reach >126 mg/dL of glucose were included in the study.^39,40^

### 5.4 Ischemia/Reperfusion Model Surgical Preparation

For the acute coronary ischemia/reperfusion infarction model, the experimental subjects were anesthetized with ketamine/xylazine 80–10 mg/kg b.w. i.p. with maintenance doses of 5–10 mg/kg, fixing a cannula to mechanically ventilate with oxygen-enriched air through a positive pressure respirator (Ugo Basile SRL). Ventilation was adjusted to maintain blood gases at 2.5–3 cm^3^ of air at 60 breaths per minute. Subsequently, the pericardium was dissected, and the left anterior descending coronary artery (LAD) was located, causing the occlusion of blood flow by clamping the artery with surgical silk. Teflon tubing was removed after 1 h to initiate myocardial tissue reperfusion.^40,41^

To assess postinfarction damage, the heart was removed at 4 h and immersed in isotonic NaCl solution at room temperature for two minutes, wrapped in a polyurethane film, and frozen at −20 °C for 2 h. Once the tissue was solid, it was cut into slices of 2 mm each, and the slices were stained with 1% triphenyltetrazolium (Sigma-Aldrich, USA) (0.5 g of 2,3,5-triphenyltetrazolium chloride in 50 mL of 100 mM phosphate buffer solution at pH 7.4), incubated at 37 °C for 20 min in a water bath (Lumistell IHA-12L), and immersed for 20 min in a solution of 4% p-formaldehyde (Sigma-Aldrich, USA) in 100 mM phosphate buffer (PBS) at pH 7.4 at room temperature. Images of the slices were digitized and processed with ImageJ software (1.30). The ratio of damage induced by coronary ischemia/reperfusion was expressed as the percentage ratio of the infarcted area over the total healthy area.^42^

### 5.5 Biochemical Analysis

To evaluate the antiprediabetic potential of the ExPhy extract administered at different doses and to compare against Am treatment at 500 mg/kg, glucose concentration was measured by a glucometer method (SD Check Biosensor, Inc.) with 12 h fasting.^43^

Blood samples were collected in gold Vacutainer tubes with a separating gel and coagulation activator and centrifuged at 3,500 rpm for 5 min to obtain blood serum. Cholesterol-LQ determinations were performed using a commercial kit (Spinreact, S.A/S.A.U, Spain).^36^ HDLc-P cholesterol was determined using a commercial kit (Spinreact, S.A/S.A.U, Spain). High-density lipoproteins were determined using the Cholesterol-LQ enzymatic reagent after precipitation of VLDL and LDL lipoproteins with phosphotungstate in the presence of magnesium ions. The determination of LDLc-D cholesterol was performed with a commercial kit (Spinreact, S.A/S.A.U, Spain) by the elimination of non-LDL lipoproteins by the action of cholesterol esterase, cholesterol oxidase, and catalase and subsequent formation of a colored compound. Finally, triglycerides (GPO-POD) were determined by means of a commercial enzymatic colorimetric kit (Spinreact, S.A/S.A.U, Spain). Triglycerides release glycerol and free fatty acids by the action of lipoprotein lipase (LPL) to produce glycerol-3-phosphate (G-3-P) and adenosine-5-diphosphate (ADP). The production of hydrogen peroxide causes red coloration, which is proportional to the concentration of the metabolite in the sample tested. All of the reagents used were of analytical grade. All determinations were performed in an UV–vis spectrophotometer (VE-5100 UV).^44^

### 5.6 Statistical Analysis

Results were analyzed by one-way ANOVA, considering significant differences when *p* ≤ 0.05 (*) and highly significant differences when *p* ≤ 0.01 (**). Post hoc analysis was performed by using the Student–Newman–Keuls test. GraphPad Prism 6.01 software was used for this analysis, and the data were represented in graphs ± SEM.

## #

G.A.C.C. deceased on December 13, 2024.
